# Current status and determinants of maternal healthcare utilization in Afghanistan: Analysis from Afghanistan Demographic and Health Survey 2015

**DOI:** 10.1371/journal.pone.0217827

**Published:** 2019-06-11

**Authors:** Sarwat Mumtaz, Jinwook Bahk, Young-Ho Khang

**Affiliations:** 1 Department of Health Policy and Management, College of Medicine, Seoul National University, Seoul, South Korea; 2 Department of Health Policy and Management, School of Public Health, University of Hail, Hail, Kingdom of Saudi Arabia; 3 Department of Public Health, Keimyung University, Daegu, South Korea; 4 Institute of Health Policy and Management, Seoul National University Medical Research Center, Seoul, South Korea; Brandeis University, UNITED STATES

## Abstract

**Background:**

Advancing maternal health is central to global health policy-making; therefore, considerable efforts have been made to improve maternal health. Still, in many developing countries, particularly in Sub-Saharan Africa and South Asia, including Afghanistan, the maternal mortality ratio (MMR) remains high. The objective of this study was to examine the determinants and current status of the utilization of maternal healthcare in Afghanistan.

**Methods:**

This study used the most recent data from the Afghanistan Demographic and Health Survey 2015. The unit of analysis for this study was women who had a live birth in the five years preceding the survey. The outcome variables were four or more antenatal care (ANC) visits, delivery assistance by a skilled birth attendant (SBA), and delivery by cesarean section (CS). The explanatory variables were basic sociodemographic characteristics of the mothers. We examined the sociodemographic characteristics of women utilizing ANC, SBA, and CS using descriptive statistics and estimated usage of ANC, SBA and CS after adjusting for maternal age and parity groups via direct standardization. Multivariable logistic regression models were employed to investigate the determinants of maternal healthcare variables.

**Results:**

Overall, 17.8% of women attended four or more ANC visits, 53.6% utilized an SBA, and 3.4% of women gave birth through CS. Women’s education, wealth status, urbanity, autonomy, and availability of their own transport were found to be the major determinants of service utilization.

**Conclusions:**

This study underscores low utilization of maternal healthcare services with wide disparities in Afghanistan and highlighted the need for an adequate health strategy and policy implementation to improve maternal healthcare uptake.

## Introduction

Advancing maternal health is central to global health policy-making; therefore, considerable efforts have been made to improve maternal health, resulting in a remarkable decline in the maternal mortality ratio (MMR) from 385 to 216 deaths per 100,000 live births between 1990 and 2015 worldwide [1]. The global burden of maternal deaths is still high in many developing countries, particularly in Sub-Saharan Africa and South Asia [2]. More than 250,000 women died during pregnancy in 2015; mostly from preventable causes ranging from sepsis and eclampsia to obstructed labor and severe bleeding. These deaths could have been avoided if those women had access to maternal healthcare services, such as antenatal care (ANC), skilled birth attendant (SBA), and emergency obstetric care [2]. Access to high-quality maternal healthcare irrespective of economic position and social group is the right of every woman around the globe [3]. Empirical evidence suggests that many developing countries, including Afghanistan, have a persistently high burden of maternal mortality with huge regional and wealth disparities in maternal healthcare utilization [2, 4, 5].

Afghanistan is a low-income country located in South-Central Asia that has been overwhelmed by wars for decades and is well known for poverty, political instability, a devastated health infrastructure, conflicts, violence, and geographical barriers. As a result, the country’s healthcare system is combating several sociodemographic and health challenges, ranging from a high fertility rate and low life expectancy in general to high maternal and child-related morbidity and mortality [6]. Although the MMR declined in Afghanistan from 1340 in 1990 to 396 in 2015, Afghanistan remains among the three countries outside the Sub-Saharan African region with the highest MMR in the world. The other two countries are Yemen (with 385 per 100,000 live births) and Haiti (with 359 per 100,000 live births) [7]. Studies suggest that socioeconomic and demographic factors play an important role in the accessibility, availability, and affordability of maternal healthcare services [8–12]. Therefore, monitoring the status and determinants of utilization of maternal healthcare may facilitate the continuous improvement of health systems and the development of equity-oriented policies.

ANC, SBA (doctors, nurses, and midwives), and the availability of emergency obstetric care are important components of basic maternal healthcare that can help reduce maternal and child mortality and provide the opportunity to pregnant women to have a safe childbirth [13]. ANC visits are crucial for ensuring best health outcomes for both mothers and children. Early and timely ANC visits provide the opportunity to detect any associated disease in women during pregnancy (e.g., anemia, eclampsia, gestational diabetes). The WHO recommended at least eight antenatal visits in new guidelines in 2016 for a positive experience and quality of life throughout the duration of pregnancy [14]. The term ‘skilled birth attendant’ was defined by a joint statement by the WHO/UNFPA/UNICEF/ World Bank in 1999 as “exclusively the people with midwifery skills (for example, doctors, midwives, nurses) who have been trained to proficiency in the skills necessary to manage normal deliveries and diagnose, manage or refer complications’. The significance of having a skilled attendant at delivery has been reported in a number of studies, yet many women in developing countries deliver outside the health facilities without a skilled attendant at childbirth [15]. Similarly, the emergency obstetric care like cesarean section is not accessible to all pregnant women in developing countries [16]. Evidences show that proper utilization of quality maternal healthcare is associated with improved maternal and neonatal outcomes in Afghanistan [5,17]. Therefore, there is a strong need to explore the possible factors that determine the maternal healthcare utilization. A number of studies have demonstrated various socioeconomic and regional factors in maternal healthcare utilization in Afghanistan [17–19]. However, these studies were largely based on a previous mortality survey or used single measures, such as education, area of residence, region, and wealth quintile. To explore the determinants of coverage of maternal healthcare, it is essential to understand the social, demographic, and structural factors associated with maternal healthcare utilization. This study aimed to analyze the data from the most recent, standardized Afghanistan Demographic and Health Survey (AfDHS) 2015, to explore the current status of utilization maternal healthcare; ANC, SBA, and cesarean section (CS) using a broad range of sociodemographic indicators, including women’s autonomy, in an effort to inform equitable policies and strategies to end preventable maternal mortality.

## Methods

### Data

This study used data from the 2015 AfDHS, a nationally representative survey conducted by the Central Statistics Organization and Ministry of Public Health Afghanistan, publicly available at https://dhsprogram.com. We downloaded the data after obtaining permission from the Demographic and Health Survey (DHS) team. The AfDHS is one of many globally authorized and publicly available DHS surveys in developing countries, funded and supported by the United States Agency for International Development. The AfDHS is the first standard demographic and health survey in the country, and collected information on a broad range of basic demographic and health indicators such as family planning, maternal and child health, the nutritional status of women and children, and knowledge and attitudes about HIV/AIDS and domestic violence. This survey employed a stratified two-stage sample design to facilitate estimates of the selected indicators at the national level, including urban areas, rural areas, and provinces. The first stage involved selecting 950 clusters (260 in urban areas and 690 in rural areas), whereas the second stage involved systematic sampling of households from a list of households in each of the sample clusters for a total sample size of 25,650 households. All ever-married women aged 15–49 in the selected households were eligible for an interview. A total of 30,434 eligible women were identified for an interview. Of these, 29,461 women were successfully interviewed, with a response rate of 97%. These women were asked questions about healthcare utilization, family planning, breastfeeding, vaccination, and domestic violence, along with sociodemographic characteristics.

The unit of analysis for this study was women who had a live birth in the five years preceding the survey. This yielded a total of 19,642 women. Our analysis was restricted to the most recent live birth. We calculated sampling weights to adjust for differences in the probability of selection and interview among respondents.

### Outcome variables

The outcome variables for this study were four or more ANC visits, delivery assistance by an SBA, and delivery by CS. Although, WHO recommended at least 8 ANC visits in new guidelines in 2016, we selected 4 or more ANC visits as our outcome variable for the study because we used 2015 DHS survey that conducted the data during five years preceding the survey. We coded ANC visits for the last delivery as ‘1’ if a woman reported four or more visits and ‘0’ for less than four visits. The SBA variable referred to delivery assistance provided by a doctor, midwife, or nurse. SBA was investigated in the 2015 AfDHS for each birth through the question ‘Who assisted with the delivery of child (NAME)?’. If the baby was delivered by a doctor, nurse, or midwife, then the SBA variable was coded as ‘yes’, otherwise as ‘no’. The last outcome variable of our study was delivery by CS. The mode of delivery was assessed with the question ‘Was (NAME) delivered by cesarean, that is, did they cut your belly open to take the baby out?’. The response was a dichotomous variable, with 1 for ‘yes’ and 0 for ‘no’.

### Explanatory variables

We selected the explanatory variables based on literature review on the determinants of maternal healthcare utilization (4,21,35,37,40,43). However, we used only those variables which were available in the 2015 AfDHS. The selected independent variables were the sociodemographic characteristics of the mothers: maternal age, parity, level of education for both mother and father, women’s current working status, urbanity (urban/rural residence), region (provinces), the availability of their own transport (car/truck), women’s decision-making authority on their husband’s earnings (women alone or jointly with their husband), and wealth index. The wealth index used for this study was constructed by the DHS team using household assets, ranging from the ownership of a television, bicycle, or car to housing characteristics, flooring materials, source of drinking water, and sanitation facilities. On the basis of this information, households were given scores. These scores were derived through principal component analysis. Then, wealth quintiles were composed by assigning these scores to each household member, dividing the distribution into five equal categories, corresponding to the poorest, poorer, middle, richer, and the richest groups [20].

### Statistical analysis

We first examined the sociodemographic characteristics of women utilizing ANC, SBA, and CS using descriptive statistics. We calculated sampling weights in order to adjust for the differences in the probability of selection and interview among respondents. We estimated usage of ANC, SBA and CS after adjusting for five-year maternal age and parity groups via direct standardization. Total samples were used as the reference population in the direct standardization. We employed multivariable logistic regression of ANC, SBA, and CS after adjusting for sociodemographic variables. The results of the multivariable logistic regression analyses are reported as odds ratios (ORs) with 95% CIs adjusted for maternal age, parity, women’s education, women’s working status, wealth index, urbanity, women’s autonomy, and transport availability. The father’s education was not included in the simultaneously adjusted model, considering the multicollinearity with women’s education. Region was not included in the logistic regression, since some categories of these variables had small sample sizes. We considered that urbanity and other socioeconomic factors reflected aspects of regional characteristics. All analyses were performed using SAS statistical software version 9.4 (SAS Institute Inc., Cary, NC, USA).

### Ethical approval

This study was based on publicly available secondary data from standard DHS survey that has been reviewed and approved by the ICF International Institutional Review Board, and informed consent was already obtained from the respondents during the survey. We obtained permission from the DHS program to use the data for this study thus no further ethical approval was required.

## Results

Table 1 shows the percent distribution of sociodemographic characteristics of women aged 15 to 49 years who had a live birth in the five years preceding the AfDHS 2015, and the use of maternal healthcare (ANC, SBA, and CS) for their most recent birth. Among 19,642 women, 54% were in their 20s and 83% had no formal education, while 21% of their husbands were educated at the secondary level. Only 31% of women had the autonomy to decide alone or jointly with their husbands about how to spend the husband’s earnings. Approximately 15% of mothers had one child, 33% had four to six children, and almost 19% had more than seven children. Three-quarters of women lived in rural areas and 19% resided in the northern region. Only 11% of women were currently working, and 14% had their own transport. Table 1 also presents the percent distribution of women aged 15 to 49 years who had a live birth in the five years preceding the AfDHS 2015, who utilized maternal healthcare (ANC, SBA, and CS) for their most recent birth, according to their sociodemographic characteristics. Of the total of 19,642 women, 17.8% had attended four or more ANC visits, 53.6% had received delivery assistance from an SBA, and 3.4% gave birth through CS for their most recent delivery. Women with an age group 40–49 years had less usage of four or more ANC visits and SBA at childbirth. No major differences according to age group were observed in the utilization of CS. The proportions of women utilizing ANC, SBA, and CS were somewhat higher (22.6%, 66.1%, and 5.1%, respectively) for the first birth than for subsequent births. The utilization of ANC, SBA, and CS increased with the educational level of both women and their husbands. Women with the highest level of education had relatively high rates of ANC (52.4%), SBA (97.5%), and CS (12.3%). Likewise, maternal healthcare utilization was more common in the richest group. Rural disadvantages in the use of ANC, SBA, and CS were also found. Approximately 32% of urban women reported four or more ANC visits, while only 13.6% of rural women did so. Similarly, the rate of SBA was 81.0% in urban areas and 45.3% in rural areas. A similar pattern was found in the utilization of CS: 8.2% of total deliveries in urban areas were conducted by CS, while only 1.9% of total deliveries were by CS in rural areas. Regional and ethnic disparities in maternal healthcare usage were also found. Higher rates of ANC, SBA, and CS (33.1%, 79.0% and 8.9%, respectively) were seen in the capital region, whereas the lowest rates of ANC (5.5%), SBA (34.9%), and CS (1.3%) were found in the southern, western, and southeastern regions, respectively.No remarkable differences were observed in the utilization of maternal healthcare in working and non-working women. Women with their own transport showed more utilization of ANC, SBA and CS (20%, 68.0% and 5%, respectively). Similarly, women with more autonomy regarding their husband’s earnings had a greater tendency to receive ANC (23%), SBA (59%) and CS (4.5%).

**Table 1:** Utilization of ANC visits, SBA and CS according to sociodemographic characteristics, among respondents *(N = 19*,*642)*.

Sociodemographic Characteristics	Total		ANC*		SBA**		CS***	
	Number	%	Number	%	Number	%	Number	%
**Women's age (years)**	**19,642**	**100.0**	**3494**	**17.8**	**10,530**	**53.6**	**665**	**3.4**
15–19	856	4.4	155	18.1	464	54.2	35	4.0
21–24	4962	25.3	914	18.4	2861	57.7	128	2.6
25–29	5609	28.6	968	17.3	2982	53.2	203	3.6
30–34	3466	17.6	602	17.4	1774	51.2	94	2.7
35–39	2975	15.1	550	18.6	1619	54.4	133	4.5
40–44	1149	5.8	193	16.9	537	46.7	45	3.9
45–49	625	3.2	112	17.8	293	46.9	27	4.4
**Parity**								
1	2983	15.2	673	22.6	1972	66.1	151	5.1
2–3	6239	31.8	1129	18.1	3463	55.5	212	3.4
4–6	6583	33.5	1079	16.4	3301	50.1	190	2.9
7+	3837	19.5	613	16.0	1794	46.7	113	2.9
**Women's education**								
No education	16288	82.9	2390	14.7	7846	48.2	436	2.7
Primary	1596	8.1	434	27.2	1180	73.9	98	6.1
Secondary	1432	7.3	500	34.9	1187	82.9	91	6.4
Higher	326	1.7	171	52.4	317	97.5	40	12.3
**Husband’s education**								
No education	11185	57.1	1584	14.2	4826	43.1	270	2.4
Primary	2867	14.6	504	17.6	1751	61.1	109	3.8
Secondary	4120	21.0	920	22.4	2841	69.0	179	4.4
Higher	1249	6.4	461	36.9	996	79.7	85	6.8
Don’t know	158	0.8	17	10.7	70	44.5	5	3.0
**Current working status**								
Not working	17383	88.9	3106	17.9	9271	53.3	558	3.2
Working	2168	11.1	371	17.1	1199	55.3	80	3.7
**Wealth index**								
Poorest	3914	19.9	409	10.5	1026	26.2	44	1.1
Poorer	3966	20.2	453	11.5	1604	40.5	90	2.3
Middle	4020	20.5	579	14.4	1910	47.5	72	1.8
Richer	4057	20.7	797	19.7	2785	68.6	147	3.6
Richest	3685	18.8	1255	34.1	3204	86.9	311	8.5
**Urbanity**								
Urban	4566	23.2	1444	31.7	3698	81.0	375	8.2
Rural	15076	76.8	2050	13.6	6832	45.3	290	1.9
**Region**								
Northern	3737	19.0	809	21.7	2168	58.0	69	1.9
North Eastern	2665	13.6	427	16.0	1164	43.7	66	2.5
Western	3000	15.3	515	17.2	1048	34.9	81	2.7
Central highland	422	2.1	84	19.9	158	37.5	12	2.8
Capital	3509	17.9	1157	33.1	2772	79.0	312	8.9
Southern	3249	16.5	180	5.5	1529	47.1	70	2.2
South Eastern	1451	7.4	113	7.8	837	57.7	18	1.3
Eastern	1609	8.2	209	13.0	852	53.0	37	2.3
**Decision-making autonomy** **about husband’s earning**								
No	12913	66.8	1968	15.3	6630	51.3	358	2.8
Yes	5933	30.7	1383	23.4	3509	59.2	268	4.5
**Own transport**								
No	16702	85.2	2901	17.4	8556	51.2	528	3.2
Yes	2768	14.1	547	19.8	1881	67.9	134	4.8

*ANC = antenatal care

**SBA = skilled birth attendant

***CS = cesarean section

All percentages are weighted, so the absolute number of participants does not perfectly correspond to percentages in some categories.

Due to missing cases in some categories, the frequencies do not correspond to the total frequencies.

Table 2 presents the maternal age- and parity-adjusted rates of ANC, SBA, and CS. In general, the patterns according to sociodemographic factors are similar to those presented in Table 1. The highest rates of the outcome variables were seen in women with a higher educational level and those in the richest wealth quintile. Similarly, higher rates of ANC, SBA, and CS were observed among urban-living women than among rural-living women. Further, the extent of access to maternal healthcare showed wide regional and ethnic disparities. Utilization of ANC, SBA, and CS differed according to women’s autonomy, while only the SBA rates significantly differed by the ability of their own transport.

**Table 2:** Maternal age- and parity-adjusted rates (95% confidence intervals) of ANC visits, SBA, and CS according to sociodemographic characteristics, using the study sample (*N* = 19,642) as the standard population for direct standardization.

Characteristics	ANC*	SBA **	CS ***
**Overall rate**	17.7 (16.2–19.1)	52.5 (49.4–55.5)	3.2 (2.7–3.8)
**Women's education**			
No education	14.8 (13.4–16.1)	47.6 (44.6–50.5)	2.6 (2.2–3.0)
Primary	27.4 (22.5–32.3)	72.9 (67.9–77.9)	6.2 (3.1–9.2)
Secondary	35.5 (27.5–43.5)	81.4 (77.8–85.0)	6.4 (3.6–9.2)
Higher	52.5 (39.2–65.7)	95.3 (93.1–97.5)	12.1 (5.8–18.5)
**Husband's education**			
No education	14.1 (12.4–15.8)	42.5 (39.1–45.9)	2.3 (1.8–2.9)
Primary	17.4 (14.4–20.4)	59.9 (55.6–64.3)	3.7 (2.3–5.1)
Secondary	22.4 (19.6–25.1)	67.8 (64.7–70.9)	4.3 (2.9–5.7)
Higher	37.7 (32.9–42.5)	78.2 (74.5–82.0)	6.7 (4.0–9.4)
**Current working status**			
Not Working	16.7 (13.1–20.3)	54.0 (48.5–59.4)	3.5 (1.9–5.2)
Working	17.8 (16.3–19.2)	52.2 (49.1–55.3)	3.1 (2.5–3.7)
**Wealth index**			
Poorest	10.3 (8.4–12.1)	25.5 (22.4–28.6)	1.0 (0.5–1.5)
Poorer	11.3 (9.0–13.7)	39.6 (36.6–42.7)	2.2 (1.4–3.0)
Middle	14.4 (11.7–17.1)	46.8 (40.8–52.8)	1.7 (1.1–2.3)
Richer	19.8 (16.8–22.7)	67.7 (64.1–71.2)	3.6 (2.4–4.7)
Richest	34.3 (29.9–38.7)	85.7 (83.1–88.2)	8.3 (6.0–10.6)
**Urbanity**			
Urban	31.8 (28.4–35.1)	79.7 (76.4–83.1)	8.1 (6.2–9.9)
Rural	13.5 (12.0–14.9)	44.4 (40.6–48.1)	1.8 (1.5–2.2)
**Region**			
Northern	34.7 (28.7–40.8)	85.4 (80.1–90.8)	10.9 (7.6–14.2)
North Eastern	27.8 (20.1–35.6)	54.0 (45.0–63.1)	1.6 (0.5–2.8)
Western	40.3 (35.4–45.3)	55.5 (46.6–64.4)	3.6 (1.7–5.4)
Central highland	32.2 (23.0–41.5)	63.8 (50.3–77.3)	3.9 (2.0–5.8)
Capital	14.9 (10.0–19.8)	75.3 (69.7–80.9)	7.1 (4.4–9.8)
Southern	25.4 (19.0–31.7)	65.6 (55.1–76.0)	2.8 (1.7–4.0)
Southeastern	14.3 (10.3–18.3)	66.1 (53.7–78.4)	3.3 (1.5–5.0)
Eastern	30.3 (19.1–41.5)	64.6(54.2–74.9)	4.0(0.5–7.5)
**Decision-making autonomy** **about husband’s earning**			
No	15.1 (13.4–16.8)	50.1 (46.1–54.1)	2.7 (2.1–3.2)
Yes	23.3 (20.8–25.8)	58.2 (55.1–61.2)	4.4 (3.2–5.6)
**Own transport**			
No	17.2 (15.7–18.7)	50.1 (46.9–53.4)	3.0 (2.5–3.5)
Yes	20.0 (16.6–23.4)	66.6 (62.9–70.3)	4.7 (3.1–6.4)

*ANC = antenatal care

**SBA = skilled birth attendant

***CS = cesarean section

Table 3 presents crude and adjusted ORs and associated (95% CIs) of the multivariable logistic regression analysis. In the model, adjusted odds ratios were obtained by simultaneously adjusted for maternal age, parity, women’s education, women’s working status, wealth index, urbanity, women’s autonomy, and transport availability. We found that maternal age and parity were independently associated with maternal healthcare utilization after adjusting for other sociodemographic variables. Maternal age was positively associated with the use of ANC, SBA, and CS, while parity was negatively associated with the three maternal healthcare indicators. Table 3 also shows independent effects of maternal education, wealth index, and urbanity on the utilization of ANC, SBA, and CS. A significant OR for four or more ANC visits was observed for women with the highest educational level (OR = 3.52; 95% CI, 2.07–5.97) as compared to women with no formal education. The odds of delivery by an SBA and CS likewise increased with women’s educational level. The likelihood of having an SBA (OR = 13.23; 95% CI, 6.39–27.37) and CS (OR = 2.22; 95% CI, 0.15–0.52) was higher among women with the highest educational level. Belonging to the richest group was strongly associated with more ANC visits (OR = 2.69; 95% CI, 1.81–3.98), more SBA usage (OR = 11.01; 95% CI, 7.26–16.70), and more CS deliveries (OR = 2.45; 95% CI, 1.19–5.05) than their poorest counterparts. Urban residency was independently associated with the utilization of CS, with an OR of 2.82 (95% CI, 1.69–4.68). Table 3 also indicates that the utilization of maternal healthcare was likewise independently associated with women’s autonomy regarding their husband’s earnings. Women with decision-making authority on how to spend their husband’s earnings were more likely to have four or more ANC visits (OR = 1.53; 95% CI, 1.23–1.91), an SBA for delivery (OR = 1.31; 95% CI, 1.12–1.52), and childbirth through CS (OR = 1.43; 95% CI, 1.01–2.01) than women with no such autonomy. Meanwhile, the three maternal healthcare indicators were not independently associated with the ownership of one’s own means of transport.

**Table 3:** Crude and Adjusted odd ratios of ANC, SBA, and CS according to sociodemographic characteristics among respondents (*N* = 19,642).

Characteristics	ANC^a^		SBA^b^		CS^c^	
	Crude OR (95%CI)	Adjusted OR (95%CI)	Crude OR (95%CI)	Adjusted OR (95%CI)	Crude OR (95% CI)	Adjusted OR (95% CI)
**Women’s age (years)**						
15–19	1.00 (Reference)	1.00 (Reference)	1.00 (Reference)	1.00 (Reference)	1.00 (Reference)	1.00 (Reference)
21–24	1.03 (0.70–1.50)	1.16 (0.77–1.73) **	1.15 (0.93–1.43) **	1.68(1.32–2.14) ***	0.42 (0.81–1.41)	0.75 (0.40–1.41)
25–29	0.94 (0.65–1.38)	1.41 (0.90–2.20) ***	0.96 (0.74–1.20)	1.97 (1.49–2.58)***	0.96 (0.65–3.26)	1.61 (0.79–3.26) **
30–34	0.96 (0.68–1.36)	1.63 (1.00–2.65) ***	0.89 (0.69–1.14)	1.97 (1.41–2.76) ***	1.56 (0.88–3.17)	1.47 (0.68–3.17) **
35–39	1.04 (0.69–1.55)	1.86 (0.97–3.54) ***	1.02 (0.81–1.27)	2.69 (1.70–4.26) ***	2.58 (1.16–5.67)	2.38 (1.11–5.12) ***
40–44	0.93 (0.60–1.43)	2.08 (1.08–4.03) ***	0.75 (0.56–1.00)	2.57 (1.58–4.13) ***	3.01(1.29–8.63)	2.87 (1.07–7.63) ***
45–49	0.98 (0.55–1.73)	2.46(1.04–5.83) ***	0.75 (0.56–1.01)	3.03 (1.96–4.68) ***	8.63 (5.81–15.33) ***	4.53 (1.81–11.33) ***
**Parity**						
1	1.00 (Reference)	1.00 (Reference)	1.00 (Reference)	1.00 (Reference)	1.00 (Reference)	1.00 (Reference)
2–3	0.89 (0.65–1.99)	0.70 (0.55–0.89)	0.86 (0.55–1.67)	0.49 (0.42–0.59)	0.66 (0.40–1.86)	0.51 (0.30–0.86) **
4–6	0.99 (0.56–1.76)	0.58 (0.44–0.76)	0.66 (0.34–1.47)	0.36 (0.28–0.47)	0.75 (0.29–1.67) **	0.35 (0.19–0.67)
7+	1.55 (0.62–0.98) ***	0.55 (0.32–0.93)	0.40 (0.30–0.65)	0.30 (0.20–0.45)	1.28 (0.15–1.52) ***	0.28 (0.15–0.52)
**Women's education**						
No education	1.00 (Reference)	1.00 (Reference)	1.00 (Reference)	1.00 (Reference)	1.00 (Reference)	1.00 (Reference)
Primary	2.14 (1.66–2.77) ***	1.78 (1.37–2.31) ***	3.06 (2.41–3.88) ***	2.32 (1.80–2.98) ***	2.54 (1.93–3.34) ***	1.89 (1.14–3.12) ***
Secondary	3.13 (2.15–4.57) ***	2.26 (1.55–3.29) ***	5.18 (4.07–6.59) ***	2.79 (2.18–3.57) ***	4.56 (3.57–5.82) ***	1.59 (1.04–2.42) ***
Higher	6.39 (3.70–9.03) ***	3.70 (2.14–6.41) ***	9.80(12.97–17.48) ***	15.52 (6.39–27.37) ***	8.26 (6.2710.89) ***	2.22 (1.11–4.42) ***
**Current working status**						
Not working	1.00 (Reference)	1.00 (Reference)	1.00 (Reference)	1.00 (Reference)	1.00 (Reference)	1.00 (Reference)
Working	0.94 (0.74–1.20) ***	0.84 (0.69–1.04) ***	1.08 (0.89–1.31) ***	1.06 (0.87–1.36) ***	0.51(0.38–0.68) ***	1.02 (0.61–1.73)
**Wealth index**						
Poorest	1.00 (Reference)	1.00 (Reference)	1.00 (Reference)	1.00 (Reference)	1.00 (Reference)	1.00 (Reference)
Poorer	1.11 (0.83–1.49)	1.14 (0.86–1.58)	1.91 (1.65–2.21)	1.95 (1.67–2.29)	1.29 (0.82–2.03) **	2.17 (1.26–3.72) ***
Middle	1.45 (1.07–1.97) ***	1.53 (1.12–2.08) ***	2.55 (1.93–3.37) ***	2.64 (2.05–3.41) ***	2.31 (1.50–3.55) ***	1.64 (0.99–2.72) ***
Richer	2.13 (1.65–2.75)***	1.89 (1.42–2.53)***	6.15 (4.93–7.67) ***	5.55 (4.37–7.05) ***	5.28 (3.60–7.73) ***	2.33 (1.37–3.94) ***
Richest	4.50 (3.48–5.82) ***	2.69 (1.81–3.98) ***	19.03(14.57–24.86) ***	11.01(7.26–16.70) ***	9.34 (6.2413.98) ***	2.45 (1.19–5.05) ***
**Urbanity**						
Rural	1.00 (Reference)	1.00 (Reference)	1.00 (Reference)	1.00 (Reference)	1.00 (Reference)	1.00 (Reference)
Urban	2.96 (2.44–3.60)***	1.61 (1.18–2.19)***	5.18 (3.97–6.76) ***	1.45 (1.02–2.07) ***	2.65 (2.13–3.31) ***	2.82 (1.69–4.68) ***
**Decision-making** **autonomy** **about husband’s earning**						
No	1.00 (Reference)	1.00 (Reference)	1.00 (Reference)	1.00 (Reference)	1.00 (Reference)	1.00 (Reference)
Yes	1.37 (1.14–1.65) ***	1.53 (1.23–1.91) ***	1.14 (0.98–1.32) ***	1.31 (1.12–1.52) ***	1.63 (1.21–2.31) ***	1.43 (1.01–2.01) **
**Own transport**						
No	1.00 (Reference)	1.00 (Reference)	1.00 (Reference)	1.00 (Reference)	1.00 (Reference)	1.00 (Reference)
Yes	0.97 (0.89–1.23)	0.88 (0.70–1.10)	1.16 (0.89–1.28)	1.20 (0.99–1.44)	1.51 (1.82–2.79)	1.21 (0.82–1.79)

^a^ANC = antenatal care

^b^SBA = skilled birth attendant

^c^CS = cesarean section

OR: odds ratio; CI: confidence interval

*P < .05

**P < .01

***P < .001

## Discussion

This study identified low levels of having four or more ANC visits, SBA, and CS, together with a suboptimal coverage of these maternal healthcare services Afghanistan. We found that overall, only 18% of women attended four or more ANC visits, 53.6% received care by an SBA, and 3.4% of recent births were delivered by CS. With this small proportion of women using maternal healthcare, overall maternal health is at risk in Afghanistan, which accentuates the need to address the circumstances influencing the current low level of utilization of maternal healthcare services [21].

The frequency of ANC visits helps in the early detection of high-risk pregnancies, allowing preventive measures to be taken before any complications occur, and ANC has therefore been advocated as a way to reduce maternal mortality in developing countries. According to our study, approximately 18% of mothers in Afghanistan attended four or more ANC visits, which does not demonstrate satisfactory progress in ANC service utilization. The adequate utilization of antenatal care depends on many factors, such as social, political, and economic status, as well as availability of health centers [22]. However, the low level of ANC coverage in Afghanistan identified in this study is not surprising, and is in line with the findings of previous studies on maternal health service utilization in Afghanistan [23].

Our findings were similar with a recent study done in Nepal, India and Sri Lanka that has shown wide differences in the use of maternal health care services in these South Asian countries where women had lower access in utilization of SBA [24]. Our analysis showed that the overall CS rate in recent births was 3.4% in Afghanistan, with huge socioeconomic disparities within the country. According to the 2015 WHO guidelines, a CS rate below 5% is considered as an inadequate level of accessibility in cases of obstetric complications that is hazardous to safe motherhood [25].

The utilization of obstetric care services from well-trained health professionals and well-equipped medical institutions is widely recognized as an important causal factor in reducing maternal mortality [26]. A literature review of CS utilization in developing countries demonstrates that various sociodemographic, cultural, and health system factors in low-income countries shape the utilization of CS, such as education, area of residence, poverty level, lack of decision-making power among women regarding their own health, the unavailability of life-saving obstetrics services, the insufficient provision of medicines and equipment in the available emergency obstetric health units, long distances from basic health units, and the lack of SBAs [26–29]. Unfortunately, all these risk factors are present in Afghanistan, where geographic barriers, lack of SBAs, and deficits in their knowledge about the proper use of drugs and supplies may well cause large obstacles to emergency obstetric care [30,31].

In order to estimate the independent effect of each variable across all sociodemographic characteristics of the respondents, we simultaneously adjusted for the variables and found that education, wealth index, urbanity, and women’s autonomy were independent determinants of maternal healthcare in Afghanistan. These findings are consistent with those of previous studies on the assessment of inequalities in maternal healthcare utilization in Afghanistan [18,19].

Our study found that education was a powerful determinant of maternal healthcare utilization. The likelihood of having four or more ANC visits, SBA, and CS increased with the mother’s educational level. These findings are consistent with previous studies that explored the positive association of educational attainment with uptake of maternal healthcare [32,33]. Empirical evidence has suggested that maternal education is among the most important determinants of ANC utilization, after controlling for other factors [34]. Our findings also indicated that the highest level of educational attainment in women and their husbands was associated with higher rates of delivery from SBAs (97% of women and 80% of husbands). The significant association of SBA utilization with education is quite evident in the present literature. Education plays an important role in women’s healthcare utilization, and may provide women with information on safe motherhood and the possible hazards of inadequate care during pregnancy. Further, education sustains healthy lifestyles and positive choices by promoting health awareness and the likelihood of seeking high-quality healthcare services. A recent study from a rural district in Ghana explored factors associated with skilled delivery services utilization, highlighting that the mother’s educational attainment was significantly associated with utilization of skilled delivery services [35].

Maternal healthcare utilization was also found to be independently associated with wealth. The richest women were more likely to report ANC visits, SBA utilization, and CS. Conversely, the lowest coverage of ANC visits, SBA, and CS was seen in women from the poorest quintile. The existing data have identified poverty as an important factor responsible for the low utilization of maternity services. Low financial resources lead to less access to maternal healthcare facilities, as poor households lack the ability to pay for the cost of transportation; thus, the accessibility of health facilities located long distances away becomes a huge barrier, specifically, these financial and transportation problems have been found to lead to the low utilization of maternal healthcare in rural areas in Afghanistan [29]. The literature suggests that several other factors also play a significant role in the use of maternal healthcare besides transportation and distance to the health facility, such as poor infrastructure, lack of services, staff shortages, and attitudes of healthcare providers [30].

Women’s autonomy, as measured by decision-making authority over how to spend their husband’s earnings, also had an important influence on maternal service utilization in our study. This effect was largely independent of other sociodemographic factors, such as education and wealth index (Table 3). The findings of this study showed that the likelihood of attending four or more ANC visits, SBA, and delivery by CS was greater in women who had decision-making autonomy regarding their husband’s earnings. Although few women had autonomy to decide about their husband’s earnings (31%), those women showed a positive association with maternal healthcare, indicating that women’s autonomy can enhance maternal healthcare utilization. Women’s autonomy has been poorly studied in developing countries; the few existing studies have suggested that utilization of maternal healthcare services is greater among more autonomous women, as measured in relation to the spending of money, than among those whose spending is controlled by other people. Thus, women’s autonomy has a positive impact on maternal and child healthcare utilization behavior [36,37]. Women's lack of autonomy is an important issue in Afghanistan, where women have restricted abilities to access and/or receive maternal and child healthcare. Most women did not have permission to go outside, and most are forbidden to be alone when in public. Husbands and in-laws are mostly the decision-makers in Afghanistan [38].

Our study found wide regional, and urban/rural disparities, with low rates of ANC visits in rural areas (13.6%), and the southern region (5.5%), as compared to relatively high coverage of ANC in urban areas (31.7%), and the capital region (33.1%). These findings have been confirmed by other studies in Afghanistan [17,18,23].

Much has been written on regional, urban/rural, and ethnic inequalities in maternal healthcare utilization in developing countries [34,39]. The exact causes of low utilization of maternal healthcare in rural and poor regions are not well known, but various cultural and economic factors such as traditional beliefs, lack of awareness of modern technology, and the inaccessibility of health services might well act as major factors contributing to the low utilization of maternal healthcare services in rural and urban areas [40–42]. All these factors contribute equally in Afghanistan; the low educational status of men and women, their cultural beliefs, and restrictions on women’s ability to access and decide upon their own healthcare have led to low utilization of maternal healthcare services despite their availability in the area [43].

Our findings showed that in households that had their own means of transport (car or truck), there was greater utilization of SBA (67.9%) and CS (4.8%) than among their counterparts who did not have their own means of transport. However, after controlling for other factors, this effect diminished. In our study, no association was observed between the working status of women and their utilization of maternal healthcare.

This study used the most recent national level data which included 29,461 women with a response rate of 97%. However, it has some limitations. First, this is a cross-sectional study measuring exposure and outcome variables at the same time, which hampers the ability to draw causal inferences. However, reverse causality (i.e., maternal healthcare utilization affecting sociodemographic factors) is not reasonably possible. Second, our analysis was restricted to the last birth that occurred during the five years preceding the survey. This is because we focused the most recent status of maternal healthcare in Afghanistan. Third, this study is based on self-reported information by respondents and may be subject to recall bias.

## Conclusions

This study highlighted the low utilization of maternal healthcare (ANC, SBA, and CS), with wide disparities among certain socioeconomic determinants of health. Women’s education, wealth status, urbanity, and autonomy were found to be the major determinants of service utilization. This underscores the need for an adequate health strategy and the implementation of policies to raise awareness among communities to improve maternal healthcare uptake through multipurpose approaches to address both societal and medical issues faced by the women of Afghanistan. Access to emergency obstetric services should be ensured in remote locations to prevent obstetric complications. In conclusion, basic and comprehensive maternal healthcare services should be received by every woman to improve maternal health.
